# Hypoxia, Oxidative Stress and Fat

**DOI:** 10.3390/biom5021143

**Published:** 2015-06-08

**Authors:** Nikolaus Netzer, Hannes Gatterer, Martin Faulhaber, Martin Burtscher, Stephan Pramsohler, Dominik Pesta

**Affiliations:** 1 Department of Sport Science, Faculty for Sports Science and Psychology, University of Innsbruck, Innsbruck 6020, Austria; E-Mails: hannes.gatterer@uibk.ac.at (H.G.); martin.faulhaber@uibk.ac.at (M.F.); martin.burtscher@uibk.ac.at (M.B.); dominik.pesta@uibk.ac.at (D.P.); 2 Hermann Buhl Institute for Hypoxia and Sleep Medicine Research, Bad Aibling 83043, Germany; E-Mail: hypoxieleiter@hermann-buhl-hypoxie.de; 3 Department Medicine, Division Sports Medicine, University Hospitals Ulm, Ulm 89081, Germany

**Keywords:** white adipose tissue, adipocytes, hypoxia, oxidative stress, cell metabolism

## Abstract

Metabolic disturbances in white adipose tissue in obese individuals contribute to the pathogenesis of insulin resistance and the development of type 2 diabetes mellitus. Impaired insulin action in adipocytes is associated with elevated lipolysis and increased free fatty acids leading to ectopic fat deposition in liver and skeletal muscle. Chronic adipose tissue hypoxia has been suggested to be part of pathomechanisms causing dysfunction of adipocytes. Hypoxia can provoke oxidative stress in human and animal adipocytes and reduce the production of beneficial adipokines, such as adiponectin. However, time-dose responses to hypoxia relativize the effects of hypoxic stress. Long-term exposure of fat cells to hypoxia can lead to the production of beneficial substances such as leptin. Knowledge of time-dose responses of hypoxia on white adipose tissue and the time course of generation of oxidative stress in adipocytes is still scarce. This paper reviews the potential links between adipose tissue hypoxia, oxidative stress, mitochondrial dysfunction, and low-grade inflammation caused by adipocyte hypertrophy, macrophage infiltration and production of inflammatory mediators.

## 1. What Do We Really Know about Oxygen Concentration in White Adipose Tissue?

**Figure 1:**
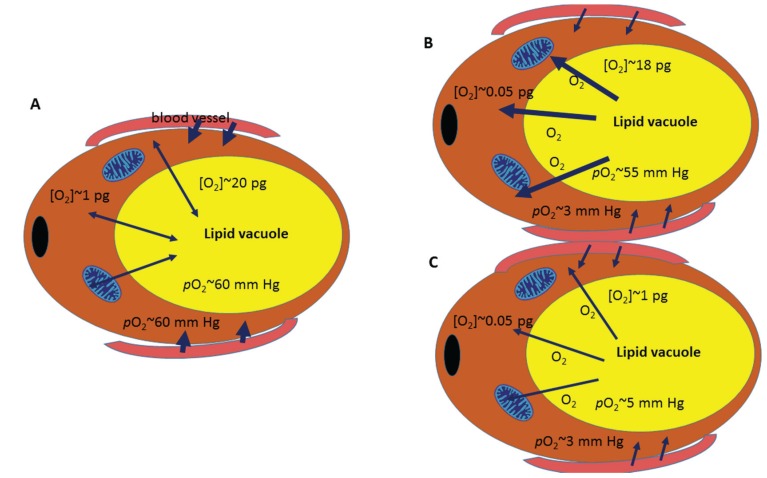
Schematic of oxygen transport in adipocytes: (**A**) Equilibrium oxygen saturation: during long-term exposure at sea level, no net flux of O_2_ occurs between the cytosolic and the lipid compartment of the cell as both compartments are in equilibrium due to saturation with O_2_. (**B**) Acute Hypoxia: during acute hypoxia, the *p*O_2_ in the cytoplasm drops whereas the *p*O_2_ in the lipid vacuole lags behind due to the slower equilibration of fat with oxygen. Due to the concentration difference, an outward flux of oxygen from the lipid vacuole into the cytoplasm is expected, which decreases with prolonged hypoxia. (**C**) Prolonged Hypoxia: with prolonged hypoxic exposure, the outward flux decreases due to faster desaturation of the cytosolic and delayed, but continuous desaturation of the lipid compartment of the cell. Assumptions are based on an adipocyte volume of 500 nL [4] and a lipid vacuole fraction of 80% of cell volume.

In order to understand under which circumstances hypoxia can induce oxidative stress in adipocytes it is useful to consider some basic physical principles. This is important because the perception of oxidative stress and fast reactions in response to hypoxia usually relate to tissues with a high blood perfusion and a solubility coefficient similar to that of water. However, this is different in fat tissue. According to William Henry’s law [1], the solubility of oxygen in fat and oil is five times higher than in water. The amount of dissolved oxygen in fat is around 5 mg/100 mL, as compared to about 0.9 mg/100 mL for water. However, according to John Scott Haldane’s [2] research about the transport of gases in blood and additional calculations of Albert Bühlmann in Zürich 50 years later [3], the saturation and desaturation of adipose tissue with oxygen, which is comparable to that of nerve and brain tissue, takes more time than that of, for example, muscle tissue. For bone, cartilage and tendon tissue, this process takes even longer. Therefore it is difficult to estimate when the effect of transient hypoxia reaches fat tissue and how fast reoxygenation can lead to oxidative stress. The partial pressure of oxygen (*p*O_2_) of white adipose tissue completely saturated with oxygen in a healthy lean young adult is 55–60 mmHg, not far from arterial blood *p*O_2_ (Figure 1A).

## 2. Hypoxia in White Adipose Tissue: A Question of Time and Dose

In their publication in the journal Circulation, Goossens and coauthors [5] show that their new method to continuously measure oxygen tension in the interstitium between adipocytes via optochemical microdialysis sensors over several hours gives a better insight into hypoxic reactions of fat cells as compared to assessment with conventional electrochemical sensors. Their results in humans differ somewhat from results obtained with electrochemical sensors, which measure *p*O_2_ at one time point only, and their results in humans differ strongly from results obtained from rodents with electrochemical sensors.

Interestingly, these authors found that, unlike what was expected, *p*O_2_ in white adipose tissue (interstitium between adipocytes) of obese individuals (80 mmHg) was higher than that in lean individuals (60 mmHg) despite a reduced arterial blood flow. This has been explained by insulin resistance and a lower oxygen consumption of already impaired adipocytes with higher inflammatory markers, such as interleukin (IL)-6, in the obese.

Nevertheless, taking these results into account, the question arises, when the hypoxic stimulus hits the fat cells and how fast changes in oxygen tension in the cell (the above mentioned measurements reflect *p*O_2_ in the interstitium) really occur. In an obese, insulin resistant individual it would take a while until a short period of hypoxia with arterial oxygen desaturation, as can be caused by obstructive sleep apnea, could transform hyperoxic or normoxic adipose tissue into a hypoxic state. However, quick changes from hyperoxia or normoxia to hypoxia would be needed in order to cause oxidative stress.

Another study by Wang *et al*. [6], on the time-dose response of adipokine secretion, in response to hypoxia in human adipocyte cell culture, also shows that exposure to hypoxia is important in order to determine if oxidative stress occurs in human fat tissue. Wang *et al*. mimicked hypoxia in the cells for 24 h using cobalt chloride (CoCl_2_) and then determined hypoxia-inducible factor (HIF)-1α as an indicator of hypoxia and leptin mRNA, adiponectin, and inflammatory markers, such as tumor necrosis factor (TNF)- α1 and IL-6, among others. As expected, HIF-1α increased, but this increase was more pronounced in preadipocytes than in adipocytes; the oxidative stress markers, inflammatory markers and leptin also increased with hypoxia while adiponectin decreased, but the time of responses differed markedly between cell types. HIF reached its maximum after 8 h during the first 24 h and then slowly decreased until day 14 [6]. The highest level of oxidative stress in accordance to the rise of the antioxidant glutathione occurred in parallel to HIF after 8 h and subsequently the adipocytes may have adapted to hypoxia and glutathione slowly decreased with HIF. Adiponectin decreased continuously right after the onset of hypoxia, whereas leptin reached its peak with a delay of 16 h. Among the inflammatory markers, which mostly go in accordance with HIF and glutathione, TNF-α already reached its peak after 2 h.

All these data demonstrate how important it is to consider the time response to hypoxia, and question acute effects in adipocytes after seconds of oxygen desaturation, as occurring, for instance, during intermittent hypoxia in obstructive sleep apnea. Not telling, however, how this type of intermittent hypoxia may have accumulative effects. In any case it has to be taken into account that after some hours of hypoxia, adipocytes start to adapt to the hypoxic stimulus accompanied by oxidative stress reduction, and that older adipocytes seem to be somewhat more “relaxed” in their reaction to hypoxia (*i.e.*, they show a delayed response) compared to younger ones.

## 3. Lipid Metabolism and Adipose Tissue Hypoxia

Metabolic disturbances in white adipose tissue in obese individuals contribute to the pathogenesis of insulin resistance and the development of type 2 diabetes mellitus. Impaired insulin action in adipocytes is associated with elevated lipolysis and increased release of free fatty acids leading to ectopic fat deposition in liver and skeletal muscle. Chronic hypoxia has been suggested to be part of pathomechanisms causing dysfunction of adipocytes [7].

In general, chronic hypoxia leads to derangements in lipid metabolism and reduced lipoprotein clearance by decreasing lipoprotein lipase activity in mice [8] and diminished subcutaneous adipose tissue lipolysis by decreased efficiency of beta-adrenergic, growth hormone and parathyroid hormone lipolytic signaling in humans [9]. A similar depression of lipolysis in human adipocytes was seen after induction of pseudo-hypoxia by ablating the adipose prolyl hydroxylase enzyme 2 gene [10]. Acute hypoxia, however, was shown to increase lipolysis by activation of adipose protein kinase A via increased epinephrine and norepinephrine release following sympathetic nervous system stimulation [11]. Propranolol treatment markedly reduced lipolysis in this experiment, suggesting that an activated sympathetic nervous system contributed to increased lipolysis. Interestingly, this is in contrast to findings from Larsen *et al*., who studied the effects of hypoxia on lipolysis in isolated rat myocardial cells and reported that propranolol did not affect the hypoxia-induced increase in lipolysis, which suggests that other, non-adrenergic mechanisms might be at play during hypoxia in cardiac myocytes [12].

In addition to its effects on lipolysis, hypoxia also alters glucose uptake capacity in human adipocytes. Wood *et al*. showed that glucose transporter (GLUT)-1, GLUT-3, and GLUT-5 gene expression and GLUT-1 protein is increased in human adipocytes in response to hypoxia, and that these changes are accompanied by a hypoxia-induced increase in glucose uptake [13]. In *ob/ob* mice, adipose tissue hypoxia caused free fatty acid release and inhibited glucose uptake in adipocytes by inhibition of the insulin-signaling pathway [14]. Thus, it seems likely that adipose tissue hypoxia will elicit different metabolic effects in non-obese and obese individuals and likely also in different types of adipose tissue, *i.e.*, central visceral (abdominal) and peripheral subcutaneous (thighs and buttocks) depots.

## 4. Blood Flow Regulation in Adipose Tissue

White adipose tissue is now recognized, not only as a passive storage organ, but also as a highly active metabolic organ. Importantly, all metabolic processes in adipose tissue depend on blood supply. Blood supply and regulation in adipose tissue differ largely from other metabolic organs, such as skeletal muscle or liver [15]. Under fasting conditions, adipose tissue blood flow is mainly regulated by vasodilatory (nitric oxide) and vasoconstrictive (alpha 2-adrenergic and angiotensin II) mechanisms, while β-adrenergic stimulation becomes important after a meal (postprandially) [15].

Disturbances in the regulation of adipose tissue blood flow have been linked to obesity and insulin-resistance [16]. It has been demonstrated that fasting adipose tissue blood flow is reduced in obese, compared to non-obese, individuals [17]. Whereas exercise training clearly increases blood flow to working muscles, this seems to not be the case for adipose tissue [15]. Since exercise training improves insulin sensitivity (mainly in skeletal muscle), but has only minor effects on adipose tissue, exercise training may improve insulin resistance independent of unaltered adipose tissue blood flow [16]. Although adipose tissue blood flow is predominantly regulated by glucose and insulin, diminished blood flow affects lipid metabolism associated with dyslipidemia in the fasting and postprandial periods [16]. It is the growing fat mass, in particular of the abdominal adipose tissue, which is associated with unfavorable changes in adipose tissue blood flow, and the development of metabolic disorders. When exposed to moderate systemic hypoxia, subcutaneous adipose blood flow at rest is comparable to that in normoxia, however, during exercise in hypoxia, adipose tissue blood flow is reduced compared to normoxic exercise [18]. This may be explained by blood flow redistribution due to the diminished availability of oxygen in skeletal muscle. Taken together, adipose tissue blood flow regulation is a very complex process, which seems to be impaired in obese and/or insulin resistant individuals. Interventions like exercise training and exposure to normobaric or hypobaric hypoxia may differently impact on adipose tissue blood flow, adipose tissue oxygen tension, oxidative stress, and related consequences.

## 5. Changes in Adipose Tissue Oxygen Tension and Its Consequences

Several studies report that hypoxia may occur within adipose tissue due to the obesity-associated expansion of adipocytes and a concomitant reduction in capillary density and blood flow [19,20,21]. However, additionally, contrasting reports exist suggesting increased [5] or unaltered oxygen content [22]. Different techniques for the determination of *p*O_2_ might be responsible for the divergent findings and it would be of importance to establish whether hypoxia or hyperoxia occurs within the adipose tissue. In various experiments, adipose tissue was exposed to extremely low or high levels of *p*O_2_, as reviewed elsewhere [23]. Both conditions lead to oxidative stress [24,25] and a pro-inflammatory response in adipocytes [23]. Oxidative stress, in addition to other factors (e.g., endoplasmic reticulum stress) was assumed to increase macrophage infiltration into white adipose tissue [26,27], thus causing further inflammation [26]. The inflammatory state induces expression of genes including TNF-α, IL-1, IL-6, monocyte chemoattractant protein-1, plasminogen activator inhibitor-1, macrophage migration inhibition factor, inducible nitric oxide synthase, matrix metalloproteinases (MMP) 9, and MMP2 [28]. The mechanistical relation of this gene expression profile is related to activation of NF-κB and HIF-1α [28]. To sum up, the oxidative stress and the pro-inflammatory state in response to severe hypoxia or hyperoxia may cause dysregulation of adipocytokines, leading to obesity-associated diseases [20,24,26]. Furthermore, the increased reactive oxygen species (ROS) secretion into peripheral blood from adipose tissue is involved in induction of insulin resistance in skeletal muscle and adipose tissue, impaired insulin secretion, and pathogenesis of various vascular diseases such as atherosclerosis and hypertension [26]. On the other hand, it was suggested that a transient increase of ROS is important for the insulin signaling pathway and might prevent further lipid storage by suppressing lipogenic genes [26]. As indicated, many findings stem from experiments performed in extremely low or high *p*O_2_. Under *in vivo* *p*O_2_ levels (3%–11% O_2_), conflicting results were found showing positive [5] or negative [29] correlations to the inflammatory status. Additionally, concentrations of 10% O_2_ and below increased adipocyte triacylglycerol content and enhanced secretion rates of IL-6 and dipeptidyl-peptidase-4 [30]. Based on the findings of Goossens *et al*. who found increased *p*O_2_ levels that positively correlated with several pro-inflammatory markers and negatively correlated with peripheral insulin sensitivity [5], one might hypothesize that chronic hypoxic exposure might positively affect the inflammatory state. In accordance with these results, mice that were exposed to chronic hypoxia for 21 days (8% O_2_) showed decreased adipocyte size, improved mitochondrial function and decreased macrophage infiltration [31]. Furthermore, in obese men, ten nights of hypoxic exposure (15% O_2_) led to increased whole-body insulin sensitivity [32]. In contrast, it should be outlined that obstructive sleep apnea syndrome, characterized by cycles of severe hypoxia, is considered a risk factor for insulin resistance [33]. Therefore, the dose, duration, and patterns of hypoxic exposure may determine the effects on metabolic and cardiovascular health.

## 6. Conclusions

Diminished blood flow and metabolic disturbances in white adipose tissue in obese individuals contribute to the pathogenesis of insulin resistance and the development of type 2 diabetes mellitus. Hyperinsulinemia leads to increased lipolysis, ectopic deposition of fat in liver and skeletal muscles decreased glucose uptake into adipocytes by inhibition of the insulin-signaling pathway.

Since vasculature cannot expand with adipocyte hypertrophy, hypoxia, mitochondrial dysfunction, and oxidative stress are important consequences, accompanied by impaired adipokine secretion and inflammation. Hypoperfusion and hypoxia in adipose tissues likely underlie the dysregulated production of adipocytokines and metabolic syndrome in obesity. Nutritional interventions, weight loss, and regular physical activity are the most promising measures to counteract these deleterious effects. Clinically, these countermeasures will likely result in a decrease in fat mass and vasculature remodeling, resulting in increased perfusion of adipose tissue, improved mitochondrial function, and metabolic health.
